# Hyperthermic intraperitoneal chemotherapy in colorectal cancer

**DOI:** 10.1093/bjsopen/zrae017

**Published:** 2024-05-09

**Authors:** Oliver M Fisher, Chris Brown, Jesus Esquivel, Stein G Larsen, Winston Liauw, Nayef A Alzahrani, David L Morris, Vahan Kepenekian, Isabelle Sourrouille, Frédéric Dumont, Jean-Jacques Tuech, Cécilia Ceribelli, Béranger Doussot, Olivia Sgarbura, Mohammed Alhosni, Francois Quenet, Olivier Glehen, Peter H Cashin, Peritoneal Surface Oncology Group International (PSOGI), Nordic Peritoneal Oncology Group (NPOG), American Society for Peritoneal Surface Malignancy (ASPSM) and BIG-RENAPE Groups, Kjersti Flatmark, Wilhelm Graf, Heikki Takala, Andrew M Lowy, Terence Chua, Joerg Pelz, Dario Baratti, Joel M Baumgartner, Richard Berri, Pedro Bretcha-Boix, Marcello Deraco, Guillermo Flores-Ayala, Alberto Gomez-Portilla, Santiago González-Moreno, Martin Goodman, Evgenia Halkia, Shigeki Kusamura, Mecker Moller, Guillaume Passot, Marc Pocard, George Salti, Armando Sardi, Maheswari Senthil, John Spilioitis, Juan Torres-Melero, Kiran Turaga, Jean-Marc Bereder, Jean-Louis Bernard, Naoual Bakrin, Sébastien Carrère, Julien Coget, Eddy Cotte, Olivier Facy, Maximiliano Gelli, François-Noël Gilly, Pablo Ortega-Deballon, Guillaume Passot, Patrick Rat, Pascal Rousset, Emilie Thibaudeau, Delphine Vaudoyer

**Affiliations:** Department of Surgery, St George Hospital, Sydney, NSW, Australia; St George & Sutherland Clinical School, UNSW Australia, Kogarah, NSW, Australia; Notre Dame University School of Medicine, Sydney, NSW, Australia; NHMRC Clinical Trials Centre, Sydney, NSW, Australia; Division of Surgical Oncology, Frederick Memorial Hospital, Frederick, Maryland, USA; Department of Surgical Oncology, The Norwegian Radium Hospital, Oslo University Hospital, Oslo, Norway; St George & Sutherland Clinical School, UNSW Australia, Kogarah, NSW, Australia; Department of Medical Oncology, St George Hospital, Sydney, NSW, Australia; Department of Surgery, St George Hospital, Sydney, NSW, Australia; Department of surgery, National Guard Health Affairs, King Abdulaziz Medical City, Riyadh, Saudi Arabia; Department of Surgery, St George Hospital, Sydney, NSW, Australia; Department of Digestive Surgery, Hôpital Hospitalier Lyon Sud, Hospices Civils de Lyon, Lyon, France; EA 3738 CICLY, Université Lyon 1, Lyon, France; Department of Surgery, Institute Gustave Roussy, Villejuif, France; Department of Oncological Surgery, Institut de Cancérologie de l’Ouest René Gauducheau, St Herblain, France; Department of Digestive Surgery, Centre Hospitalo-Universitaire de Rouen, Rouen, France; Department of Surgery, Centre Hospitalo-Universitaire l’Archet II, Nice, France; Department of Digestive Surgery, Centre Hospitalo-Universitaire Dijon Bourgogne, Dijon, France; IRCM, Institut de Recherche en Cancérologie de Montpellier, INSERM U1194, Institut régional du Cancer de Montpellier, Université de Montpellier, Montpellier, France; Département de Chirurgie Oncologique, Institut régional du Cancer de Montpellier, Montpellier, France; Surgical Oncology Division, Department of Surgery, Sultan Qaboos University Hospital SQUH, Muscat, Oman; Département de Chirurgie Oncologique, Institut régional du Cancer de Montpellier, Montpellier, France; Department of Digestive Surgery, Hôpital Hospitalier Lyon Sud, Hospices Civils de Lyon, Lyon, France; EA 3738 CICLY, Université Lyon 1, Lyon, France; Department of Surgical Sciences, Uppsala University, Uppsala, Sweden; Department of Surgery, Akademiska Sjukhuset, Uppsala, Sweden

## Abstract

**Background:**

This study evaluated the efficacy of hyperthermic intraperitoneal chemotherapy (HIPEC) in colorectal cancer with peritoneal metastases (pmCRC) in a large international data set of patients.

**Patients and Methods:**

Patients with pmCRC from 39 centres who underwent cytoreductive surgery with HIPEC between 1991 and 2018 were selected and compared for the HIPEC protocols received—oxaliplatin-HIPEC *versus* mitomycin-HIPEC. Following analysis of crude data, propensity-score matching (PSM) and Cox-proportional hazard modelling were performed. Outcomes of interest were overall survival (OS), recurrence-free survival (RFS) and the HIPEC dose–response effects (high *versus* low dose, dose intensification and double drug protocols) on OS, RFS and 90-day morbidity. Furthermore, the impact of the treatment time period was assessed.

**Results:**

Of 2760 patients, 2093 patients were included. Median OS was 43 months (95% c.i. 41 to 46 months) with a median RFS of 12 months (95% c.i. 12 to 13 months). The oxaliplatin-HIPEC group had an OS of 47 months (95% c.i. 42 to 53 months) *versus* 39 months (95% c.i. 36 to 43 months) in the mitomycin-HIPEC group (*P* = 0.002), aHR 0.77, 95% c.i. 0.67 to 0.90, *P* < 0.001. The OS benefit persisted after PSM of the oxaliplatin-HIPEC group and mitomycin-HIPEC group (48 months (95% c.i. 42 to 59 months) *versus* 40 months (95% c.i. 37 to 44 months)), *P* < 0.001, aHR 0.78 (95% c.i. 0.65 to 0.94), *P* = 0.009. Similarly, matched RFS was significantly higher for oxaliplatin-HIPEC *versus* others (13 months (95% c.i. 12 to 15 months) *versus* 11 months (95% c.i. 10 to 12 months, *P* = 0.02)). High-dose mitomycin-HIPEC protocols had similar OS compared to oxaliplatin-HIPEC. HIPEC dose intensification within each protocol resulted in improved survival. Oxaliplatin + irinotecan-HIPEC resulted in the most improved OS (61 months (95% c.i. 51 to 101 months)). Ninety-day mortality in both crude and PSM analysis was worse for mitomycin-HIPEC. There was no change in treatment effect depending on the analysed time period.

**Conclusions:**

Oxaliplatin-based HIPEC provided better outcomes compared to mitomycin-based HIPEC. High-dose mitomycin-HIPEC was similar to oxaliplatin-HIPEC. The 90-day mortality difference favours the oxaliplatin-HIPEC group. A trend for dose–response between low- and high-dose HIPEC was reported.

## Teaser

This is a large multicentric global study combining a number of large national peritoneal surface oncology databases from three continents (39 centres) for evaluating the use and choice of hyperthermic intraperitoneal chemotherapy (HIPEC) in patients with peritoneal metastasized colorectal cancer undergoing cytoreductive surgery. It is the largest study in the field of peritoneal surface oncology for colorectal cancer with crude survival analysis in over 2000 patients and propensity score matched survival analysis in over 1300 patients. This study found that oxaliplatin-based HIPEC resulted in superior overall and recurrence-free survival compared to mitomycin C-based HIPEC—a finding which persisted after propensity score matching. However, high-dose mitomycin C protocols resulted in improved survival, and a dose–response relationship between low-dose and high-dose HIPEC treatment regardless of protocol drug used was further discovered.

## Introduction

Colorectal cancer is the third most frequent malignancy in men and women and the second highest cause of cancer-related mortality worldwide^1^. Peritoneal metastases are detected in approximately 30% of patients with metastatic or recurrent disease^2^. Cytoreductive surgery (CRS) and the intraoperative instillation of hyperthermic intraperitoneal chemotherapy (HIPEC) has resulted in median overall survival (OS) times of up to 41 months in randomized trials and prospective series from expert centres^3^, which compares favourably to the median of 16 months for systemic therapy alone^2^. The recently reported UNICANCER phase III trial of oxaliplatin-HIPEC (Ox-HIPEC) for peritoneal metastasized colorectal cancer (pmCRC; PRODIGE 7) determined that there was no added survival benefit when adding HIPEC to CRS, while the 60-day complication rate was higher^3^. Equally, there exists no consensus as to which HIPEC agent should be utilized for these patients as there are a number of different protocols available^4^.

Since the reporting of the RCT on cytoreduction and hyperthermic intraperitoneal chemotherapy *versus* systemic chemotherapy and palliative surgery in patients with pmCRC^5^, many centres have utilized a mitomycin C (MMC-) HIPEC protocol; however, following additional reports^6–8^, others have adopted Ox-HIPEC. After publication of results from the PRODIGE 7 trial^9^, which applied Ox-HIPEC but failed to identify a significant survival effect, many centres have changed to MMC-HIPEC. However, currently there are significant debates concerning HIPEC use in colorectal cancer^10^.

The aim of this study was to evaluate the efficacy of HIPEC in colorectal cancer in a large international data set of patients in relation to overall and recurrence-free survival, HIPEC intensity effects and morbidity.

## Materials and methods

### Databases and study population

**Fig. 1:**
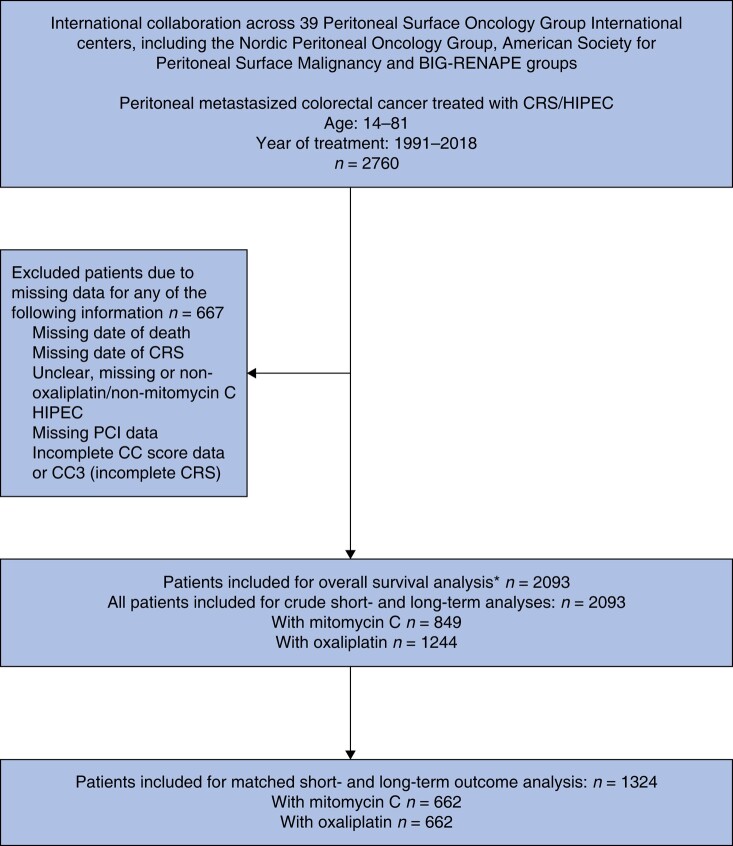
Flowchart of patient data selection and analysis

The MitOxHIPEC study (Mitomycin C *versus* Oxaliplatin Hyperthermic Intraperitoneal Chemotherapy combined with Cytoreductive Surgery for the Treatment of Colorectal Cancer with Peritoneal Metastases) was a retrospective, multi-institutional cohort study investigating the management of patients who underwent CRS/HIPEC for the treatment of pmCRC. Data were compiled across eight countries and 39 treatment centres that were part of the Peritoneal Surface Oncology Group International (PSOGI), Nordic Peritoneal Oncology Group (NPOG), American Society for Peritoneal Surface Malignancy (ASPSM) and BIG-RENAPE Groups. Data were identified from the prospectively maintained databases of the collaborative groups and compiled in a central, standardized, de-identified and password-protected database. During inputting of data into the central database, data were checked for accuracy and completeness with potential duplicate values removed by cross-referencing patients by country and treatment centre, patient age, gender, date of CRS and corresponding intraoperative peritoneal cancer indexes (PCIs). Attempts to rectify missing or inconsistent data were made by e-mail exchanges with referral centres where possible. To ensure concordance between databases and data integrity, final data capture was audited by two of the investigators prior to analysis (*Fig. 1*). All data were collected in accordance with each institution’s guidelines for retrospective studies. The retrospective capture of data for this study was approved by each centre’s or national registry’s respective ethical review boards.

*Of these, 334 had incomplete data on recurrence. Missing data overlapped in some instances for the same patients, accordingly final number with complete data for analysis was 2093. CRS, cytoreductive surgery; HIPEC, hyperthermic intraperitoneal chemotherapy; PCI, peritoneal cancer index; CC, completeness of cytoreduction.

### Surgical procedures and utilized HIPEC protocols

The study included patients who underwent complete cytoreduction with HIPEC with either MMC- or oxaliplatin-based treatment protocols for the treatment of patients with pmCRC between 1991 and 2018. The extent of peritoneal deposits was calculated according to the PCI. CRS included the resection of the primary tumour in the context of synchronous metastatic disease with greater omentectomy, and resection of all peritoneal deposits by combining peritonectomy procedures and resections of involved organs where necessary. Completeness of cytoreduction (CC) was evaluated using the CC score^11^. The present study excluded patients who did not have complete data on their CC score or if they had macroscopic residual disease >2.5 cm (CC-3) and all analyses were adjusted for remaining CC score subgroups. HIPEC was delivered at the end of surgical procedures with protocols varying according to the institution, and HIPEC being performed using open or closed techniques, depending on unit preference. Details regarding utilized HIPEC protocols are provided in *Supplementary Methods S1*.

### Study outcomes

The primary outcome of the study was patient OS defined as time from CRS/HIPEC to date of last follow-up with death from any cause. Only patients with complete data were included in the survival analyses. Secondary outcomes included patient recurrence-free survival (RFS), with RFS defined as time from CRS to recurrence at any site or death, whichever occurred first. Survival subgroup analyses included 1-, 3- and 5-year survival rates, the impact of varying HIPEC protocols including dosages on OS/RFS as well as the impact of treatment time periods on primary endpoints. Details regarding HIPEC protocols and resulting stratifications are provided in the *Supplementary Methods*.

Further secondary outcomes included the recurrence site, in-hospital and 90-day postoperative morbidity, defined using the Clavien–Dindo classification^12^.

### Data and statistical analyses

Patient characteristics were reported using frequency and descriptive analyses. Comparison of normally distributed variables was performed using Student’s *t* test and ANOVA. Non-normally distributed data were analysed using the Kruskal–Wallis test. Categorical variables were analysed using the chi-square test if suspected 2 × 2 table cell counts were >5 and/or not more than 20% of cell counts were <5. If this assumption was not met, then the Fisher’s exact test was used.

To correct for potential treatment allocation bias and confounding factors between treatment groups, propensity score matching (PSM) was performed to control for patient baseline characteristics. In a first step, unmatched patient demographics were cross-tabulated and inspected for pre-existing imbalances of preoperative factors that may have impacted the allocation to a particular HIPEC treatment arm. Subsequently, a propensity score was calculated for each patient as the predicted probability of the allocation to Ox-HIPEC using multivariable logistic regression, which included pre- and intraoperative factors identified from the first crude group comparison supplemented by the addition of factors that may impact patient prognosis including age, gender, ASA category, the presence of positive lymph nodes, the presence of liver metastases at the time of CRS/HIPEC, PCI, CC scores and the use of preoperative systemic chemotherapy. As complete propensity scores cannot be calculated in the presence of missing data for any of the included predicting variables, missing data were computed as such to allow for the lowest attrition rate of study subjects during matching steps. Matching occurred at a 1:1 ratio with no reuse of study subjects using a nearest-neighbour method, and treatment and control units were matched in a random order to ensure optimal distribution of propensity scores. Covariate balance following matching procedures was assessed with Love plots determining mean differences and the difference in cumulative density between groups using the Kolmogorov–Smirnov tests^13^.

Methodological details are provided in *Supplementary Methods*. Overall survival and RFS times were plotted stratified according to the utilized HIPEC drug and associated dose-intensification protocols using the Kaplan–Meier method and resulting survival times compared using the log-rank test. This was first done by analysing crude (unmatched) survival rates and subsequently the matched cohorts. Uni- and multivariable Cox regression analysis was performed to assess the prognostic impact of HIPEC for both OS and RFS. The proportional hazards assumption was checked for all variables using scaled Schoenfeld residuals and by inspecting the resulting time-dependent plots. Multivariable analyses were adjusted for factors (age, gender, positive lymph node status in the primary tumour, synchronous *versus* metachronous disease, colonic *versus* rectal primaries, the presence of liver metastases, preoperative systemic chemotherapy, PCI, CC score, HIPEC agent used, HIPEC intensity protocol used, postoperative complications, the use of adjuvant chemotherapy) known to influence the prognosis of CRC patients undergoing CRS/HIPEC and were performed in the crude and matched cohorts. The crude data analysis included the computation of results for missing variables to assess their possible impact on outcomes. For matched data, no missing variables were present as this is a result of the matching process itself. Resulting data are reported as HR with associated 95% c.i. All statistical analyses were performed using R Statistical Packages and all *P* < 0.05 were considered statistically significant.

## Results

### Patient characteristics

In total, 2760 individual patients contributed to the original database. After application of selection criteria, 2093 study subjects remained, of whom 849 had MMC-HIPEC and 1244 had Ox-HIPEC (*Fig. 1*). A summary of patients’ baseline characteristics and differences between groups is provided in *Table S1*. Following PSM, 1324 patients were successfully matched (662 patients in the MMC-HIPEC and 662 patients in the Ox-HIPEC treatment group). Matching resulted in excellent covariate balance and correction of differences in underlying patient characteristics (*Fig. S1*). The mean number of patients per centre contributing to the unmatched cohort was 32 (s.d. 52.9) and matched cohort 60 (s.d. 92.8), respectively. The mean number of patients per centre per year (according to the number of years that the said centre has been included) was 10.4. An overview of matched patient characteristics is provided in *Table 1*.

**Table 1.** Propensity-matched patient demographics

Variable	Total (*n* = 1324)	Mitomycin C (*n* = 662)	Oxaliplatin (*n* = 662)	*P*
**Age**				
Median (i.q.r.)	59.0 (15.0)	59.0 (16.0)	59.0 (15.0)	0.622
**Sex**				
Female	770 (58.2)	385 (58.2)	385 (58.2)	1
Male	554 (41.8)	277 (41.8)	277 (41.8)	
**American Society for Anaesthesiologists (ASA) category**				
1	113 (8.5)	51 (7.7)	62 (9.4)	0.727
2	483 (36.5)	245 (37.0)	238 (36.0)	
3	103 (7.8)	50 (7.6)	53 (8.0)	
4	1 (0.1)	1 (0.2)	0 (0.0)	
Missing	624 (47.1)	315 (47.6)	309 (46.7)	
**Positive lymph nodes**				
No	323 (24.4)	161 (24.3)	162 (24.5)	0.893
Yes	868 (65.6)	437 (66.0)	431 (65.1)	
Missing	133 (10.0)	64 (9.7)	69 (10.4)	
**Liver metastasis**				
No	1123 (84.8)	562 (84.9)	561 (84.7)	1
Yes	191 (14.4)	95 (14.4)	96 (14.5)	
Missing	10 (0.8)	5 (0.8)	5 (0.8)	
**Synchronous vs metachronous disease**				
Synchronous	640 (48.3)	312 (47.1)	328 (49.5)	<0.001
Metachronous	621 (46.9)	289 (43.7)	332 (50.2)	
Missing	63 (4.8)	61 (9.2)	2 (0.3)	
**Preoperative systemic chemotherapy**				
No	397 (30.0)	200 (30.2)	197 (29.8)	0.957
Yes	900 (68.0)	448 (67.7)	452 (68.3)	
Missing	27 (2.0)	14 (2.1)	13 (2.0)	
**Peritoneal cancer index (PCI)**				
Median (i.q.r.)	8.0 (9.0)	8.0 (10.0)	8.0 (9.0)	0.586
**Completeness of cytoreduction (CC) score**				
CC-0	1218 (92.0)	610 (92.1)	608 (91.8)	0.964
CC-1	68 (5.1)	34 (5.1)	34 (5.1)	
CC-2	12 (0.9)	5 (0.8)	7 (1.1)	
Missing	26 (2.0)	13 (2.0)	13 (2.0)	
**HIPEC intensity**				
Single drug/low dose	200 (15.1)	167 (25.2)	33 (5.0)	<0.001
Single drug/high dose	793 (59.9)	386 (58.3)	407 (61.5)	
Double drug	227 (17.1)	22 (3.3)	205 (31.0)	
Missing	104 (7.9)	87 (13.1)	17 (2.6)	
**Second HIPEC drug**				
No second drug	1097 (82.9)	640 (96.7)	457 (69.0)	<0.001
Cisplatin	10 (0.8)	10 (1.5)	0 (0.0)	
Irinotecan	217 (16.4)	12 (1.8)	205 (31.0)	
Missing	0 (0.0)	0 (0.0)	0 (0.0)	
**Perfusion time (minutes)**				
Median (i.q.r.)	30.0 (60.0)	90.0 (30.0)	30.0 (0.0)	<0.001
**IV 5-FU during HIPEC**				
Yes	178 (13.4)	155 (23.4)	23 (3.5)	<0.001
No	570 (43.1)	1 (0.2)	569 (86.0)	
Missing	576 (43.5)	506 (76.4)	70 (10.6)	
**Adjuvant systemic chemotherapy**				
Yes	422 (31.9)	198 (29.9)	224 (33.8)	0.003
No	516 (39.0)	243 (36.7)	273 (41.2)	
Missing	386 (29.2)	221 (33.4)	165 (24.9)	
**Colonic or rectal primary**				
Colon	1193 (90.1)	608 (91.8)	585 (88.4)	0.104
Rectum	116 (8.8)	48 (7.3)	68 (10.3)	
Missing	15 (1.1)	6 (0.9)	9 (1.4)	
**Signet ring cell pathology**				
No	609 (46.0)	283 (42.7)	326 (49.2)	0.008
Yes	40 (3.0)	15 (2.3)	25 (3.8)	
Missing	675 (51.0)	364 (55.0)	311 (47.0)	
**Postoperative complications**				
< grade 3-4	813 (61.4)	387 (58.5)	426 (64.4)	0.083
>= grade 3-4	477 (36.0)	256 (38.7)	221 (33.4)	
Missing	34 (2.6)	19 (2.9)	15 (2.3)	
**In hospital or 90-day mortality**				
Yes	1244 (94.0)	610 (92.1)	634 (95.8)	0.008
No	80 (6.0)	52 (7.9)	28 (4.2)	
**Operating time (minutes)**				
Median (i.q.r.)	367.5 (180.0)	368.0 (180.0)	366.0 (169.8)	0.496
**Estimated blood loss (mls)**				
Median (i.q.r.)	300.0 (600.0)	300.0 (550.0)	300.0 (550.0)	0.003
**Treatment year**				
1991-2001	41 (3.1)	26 (3.9)	15 (2.3)	0.001
2002-2006	130 (9.8)	79 (11.9)	51 (7.7)	
2007-2011	335 (25.3)	141 (21.3)	194 (29.3)	
2012-2018	818 (61.8)	416 (62.8)	402 (60.7)	

Values are *n* (%) unless otherwise specified. HIPEC, hyperthermic intraperitoneal chemotherapy.

### Long-term survival outcomes

Of the 2093 patients included in the analysis, 931 (44.5%) died during follow-up and 1759 had complete data on recurrences. Of these, 1257 (71.5%) recurred during follow-up. Median OS was 43 months (95% c.i. 41 to 46 months) with a median RFS of 12 months (95% c.i. 12 to 13 months).

**Fig. 2:**
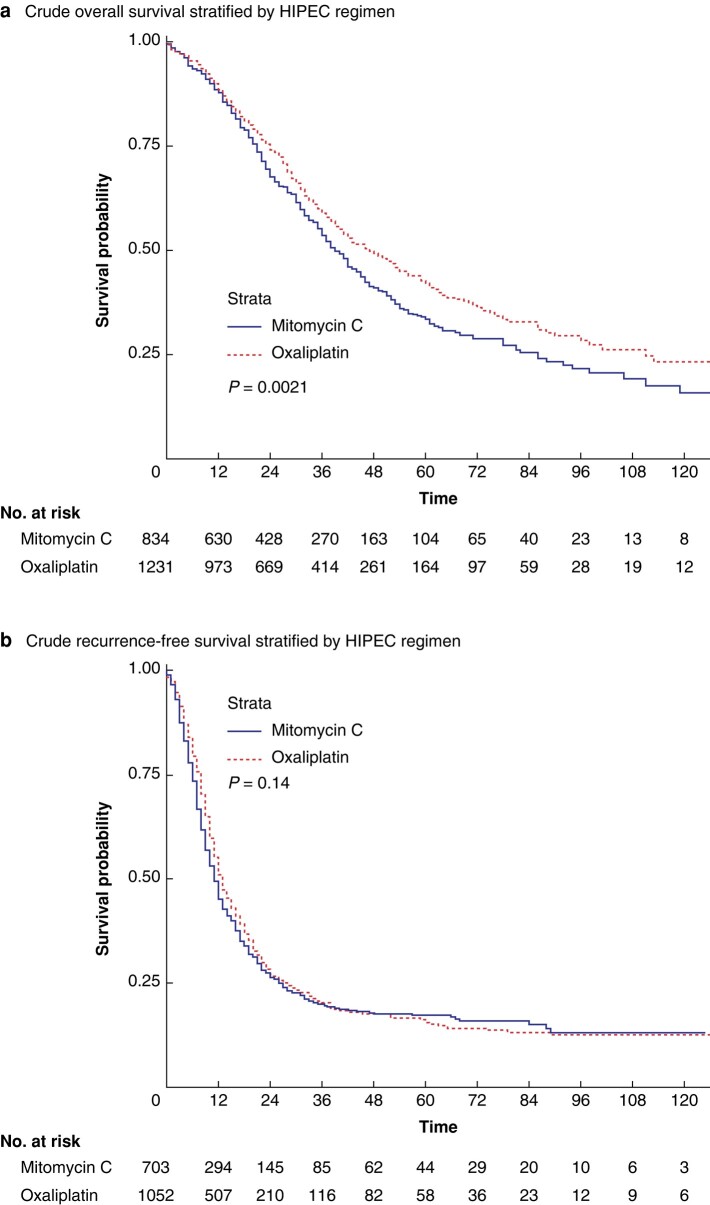
**a Crude overall survival stratified by MMC- or Ox-HIPEC**. **b Crude recurrence-free survival stratified by MMC- or Ox-HIPEC**

**Fig. 3:**
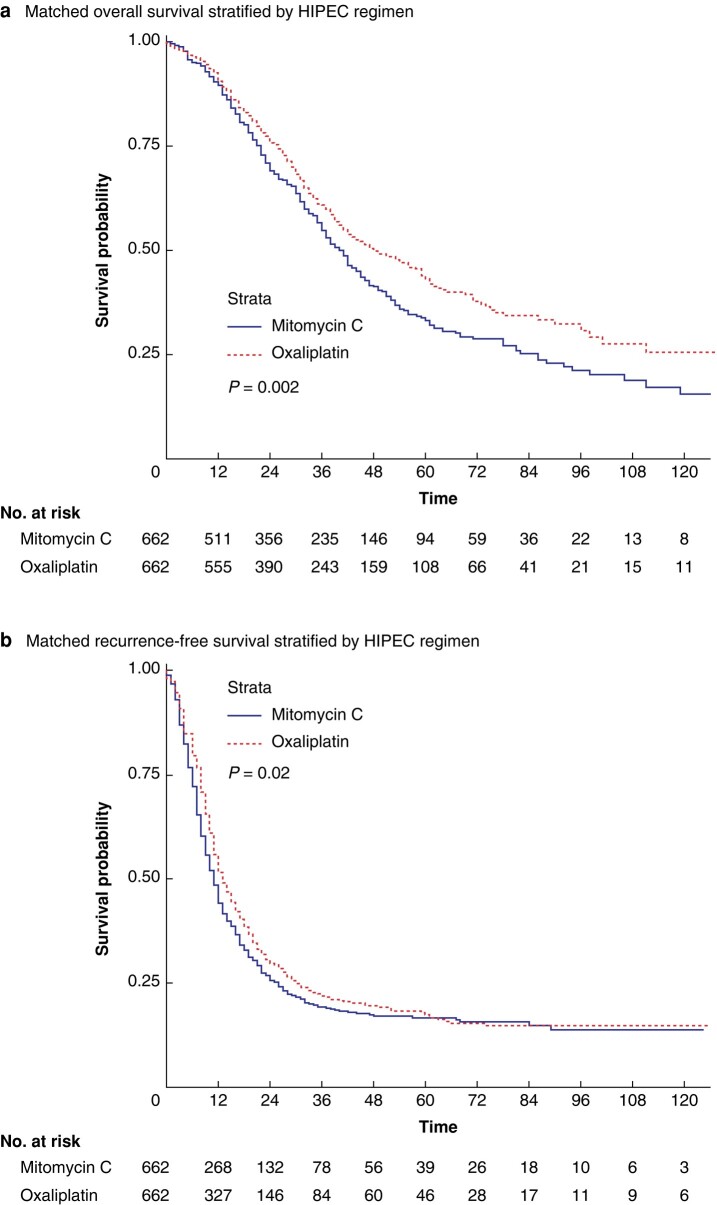
**a Matched overall survival stratified by MMC- or Ox-HIPEC**. **b Matched recurrence-free survival stratified by MMC- or Ox-HIPEC**

Median OS was significantly higher in the Ox-HIPEC group (47 months (95% c.i. 42 to 53 months) *versus* 39 months (95% c.i. 36 to 43 months), *P* < 0.001, *Fig. 2*) with a 1- and 5-year survival of 88.6% and 42.2% *versus* 87.9% and 33.6% for patients receiving MMC-HIPEC, respectively (adjusted HR (aHR) 0.77, 95% c.i. 0.67 to 0.90, *P* < 0.001). This effect persisted after PSM, with a median OS of 48 months (95% c.i. 42 to 59 months) for Ox-HIPEC *versus* 40 months (95% c.i. 37 to 44 months, *P* = 0.002, *Fig. 3*) for MMC-HIPEC. The corresponding 1- and 5-year survival was 90.6% and 43.2% *versus* 89.5% and 33.1%, respectively (aHR 0.78, 95% c.i. 0.65 to 0.94, *P* = 0.009). A summary of the uni- and multivariable Cox regression analysis of the independent effect of HIPEC regimens on OS can be found in *Table 2*. The prognostic effect of Ox-HIPEC on OS across relevant patient subgroups is depicted in a Forrest plot (*Fig. S2*).

HIPEC, hyperthermic intraperitoneal chemotherapy, MMC, mitomycin C.

HIPEC, hyperthermic intraperitoneal chemotherapy, MMC, mitomycin C.

**Table 2:** Uni- and multivariable Cox regression analysis for the impact of HIPEC regimen on overall survival in unmatched and propensity score matched patient cohorts

	Unmatched cohort (*n* = 2093)		Matched cohort (*n* = 1324)	
Variable	HR (univariable)	HR (multivariable)	HR (univariable)	HR (multivariable)
Age (years), mean(s.d.)	1.00(0.99–1.00, *P* = 0.066)	1.00(0.99–1.00, *P* = 0.297)	0.99(0.99–1.00, *P* = 0.064)	1.00(0.99–1.00, *P* = 0.504)
**Sex**				
Female	–	–	–	–
Male	1.09 (0.96–1.24, *P* = 0.173)	1.03 (0.90–1.18, *P* = 0.678)	1.11 (0.94–1.30, *P* = 0.215)	1.03 (0.88–1.22, *P* = 0.688)
**Positive lymph nodes**				
No	–	–	–	–
Yes	1.44 (1.22–1.70, *P* < 0.001)	1.41 (1.19–1.67, *P* < 0.001)	1.44 (1.18–1.76, *P* < 0.001)	1.40 (1.14–1.72, *P* = 0.001)
Missing	1.25 (0.95–1.63, *P* = 0.111)	1.11 (0.85–1.47, *P* = 0.442)	1.11 (0.80–1.54, *P* = 0.537)	0.94 (0.67–1.31, *P* = 0.708)
**Synchronous *versus* metachronous disease**				
Synchronous	–	–	–	–
Metachronous	0.95 (0.83–1.08, *P* = 0.421)	1.05 (0.92–1.21, *P* = 0.448)	0.88 (0.75–1.04, *P* = 0.132)	1.02 (0.86–1.21, *P* = 0.803)
Missing	0.77 (0.53–1.13, *P* = 0.181)	0.80 (0.53–1.20, *P* = 0.283)	1.02 (0.68–1.53, *P* = 0.909)	1.00 (0.65–1.55, *P* = 0.983)
**Colonic or rectal primary**				
Colon	–	–	–	–
Rectum	1.15 (0.92–1.44, *P* = 0.220)	1.17 (0.93–1.47, *P* = 0.182)	1.15 (0.88–1.50, *P* = 0.313)	1.12 (0.85–1.47, *P* = 0.414)
Missing	1.29 (1.04–1.60, *P* = 0.019)	1.01 (0.58–1.77, *P* = 0.972)	-	-
**Liver metastasis**				
No	-	-	-	-
Yes	1.29 (1.09–1.54, *P* = 0.004)	1.32 (1.10–1.58, *P* = 0.002)	1.32 (1.05–1.65, *P* = 0.017)	1.48 (1.17–1.88, *P* = 0.001)
Missing	0.90 (0.29–2.80, *P* = 0.852)	1.18 (0.37–3.72, *P* = 0.778)	-	-
**Preoperative systemic chemotherapy**				
No	–	–	–	–
Yes	1.01 (0.87–1.17, *P* = 0.893)	1.13 (0.97–1.33, *P* = 0.119)	0.91 (0.77–1.08, *P* = 0.277)	1.02 (0.86–1.23, *P* = 0.790)
Missing	0.77 (0.56–1.06, *P* = 0.103)	0.94 (0.67–1.31, *P* = 0.713)	0.71 (0.39–1.31, *P* = 0.273)	1.01 (0.54–1.87, *P* = 0.987)
**Peritoneal cancer index, mean(s.d.)**	1.07(1.06–1.07, *P* < 0.001)	1.06(1.05–1.07, *P* < 0.001)	1.07(1.06–1.08, *P* < 0.001)	1.06(1.05–1.07, *P* < 0.001)
**Completeness of cytoreduction score**				
CC-0	–	–	–	–
CC-1	2.47 (1.94–3.14, *P* < 0.001)	1.49 (1.15–1.92, *P* = 0.002)	2.84 (2.12–3.81, *P* < 0.001)	1.85 (1.36–2.53, *P* < 0.001)
CC-2	3.20 (2.11–4.85, *P* < 0.001)	1.94 (1.25–3.01, *P* = 0.003)	4.14 (2.14–8.02, *P* < 0.001)	2.40 (1.20–4.79, *P* = 0.013)
Missing	0.73 (0.40–1.32, *P* = 0.298)	0.66 (0.35–1.25, *P* = 0.202)	0.97 (0.50–1.87, *P* = 0.921)	1.08 (0.50–2.34, *P* = 0.843)
**HIPEC agent**				
Mitomycin C	–	–	–	–
Oxaliplatin	0.82 (0.72–0.93, *P* = 0.002)	0.77 (0.67–0.90, *P* = 0.001)	0.78 (0.66–0.91, *P* = 0.002)	0.78 (0.65–0.94, *P* = 0.009)
**HIPEC intensity**				
Single drug/low dose	–	–	–	–
Single drug/high dose	0.69 (0.57–0.83, *P* < 0.001)	0.79 (0.65–0.97, *P* = 0.025)	0.68 (0.56–0.83, *P* < 0.001)	0.74 (0.60–0.93, *P* = 0.008)
Double drug	0.59 (0.47–0.74, *P* < 0.001)	0.74 (0.57–0.97, *P* = 0.027)	0.53 (0.40–0.70, *P* < 0.001)	0.65 (0.48–0.90, *P* = 0.009)
Missing	0.85 (0.67–1.08, *P* = 0.186)	0.68 (0.47–0.99, *P* = 0.044)	0.74 (0.50–1.10, *P* = 0.140)	0.64 (0.42–0.97, *P* = 0.034)
**Postoperative complications**				
<Grade 3–4	–	–	–	–
≥Grade 3–4	1.54 (1.34–1.77, *P* < 0.001)	1.49 (1.29–1.73, *P* < 0.001)	1.58 (1.34–1.85, *P* < 0.001)	1.47 (1.24–1.74, *P* < 0.001)
Missing	1.35 (1.10–1.65, *P* = 0.004)	1.16 (0.75–1.78, *P* = 0.513)	0.95 (0.57–1.57, *P* = 0.835)	0.87 (0.48–1.58, *P* = 0.654)
**Adjuvant systemic chemotherapy**				
No	–	–	–	–
Yes	0.75 (0.63–0.88, *P* = 0.001)	0.78 (0.66–0.93, *P* = 0.006)	0.76 (0.62–0.92, *P* = 0.006)	0.81 (0.65–0.99, *P* = 0.040)
Missing	0.98 (0.84–1.15, *P* = 0.845)	0.93 (0.77–1.12, *P* = 0.454)	1.01 (0.84–1.23, *P* = 0.893)	0.92 (0.74–1.13, *P* = 0.423)

Effect estimates for other variables of interest must be interpreted with caution, due to the risk of so-called ‘Table 2 fallacy’^14^.

The median RFS for Ox-HIPEC was 13 months (95% c.i. 12 to 14 months) *versus* 11 months (95% c.i. 10 to 12 months, *P* = 0.10, *Fig. 2b*) for MMC-HIPEC, equating to a 1- and 5-year RFS of 51.0% and 15.7% *versus* 45.1% and 17.3% (aHR 0.89, 95% c.i. 0.77 to 1.02, *P* = 0.092). Following PSM, the median RFS was significantly higher for Ox-HIPEC at 13 months (95% c.i. 12 to 15 months) *versus* 11 months (95% c.i. 10 to 12 months, *P* = 0.02, *Fig. 3b*). The corresponding 1- and 5-year RFS was 51.6% and 17.4% *versus* 44.1% and 16.6%, respectively (aHR 0.84 95% c.i. 0.72 to 0.98, *P* = 0.025). A summary of prognostic factors for RFS is provided in *Table S2* and the effect of Ox-HIPEC across relevant subgroups is provided in *Fig. S3*.

### In-hospital morbidity and 90-day mortality

Major postoperative morbidity did not differ between MMC-HIPEC and Ox-HIPEC in the matched cohort, but it did differ in the unmatched cohort by only 3% (*Table 1* and *Table S1*). Ninety-day mortality differed in both the matched (MMC-HIPEC 7.9% *versus* Ox-HIPEC 4.2%, *P* = 0.008) and unmatched cohorts (MMC-HIPEC 9.0% *versus* Ox-HIPEC 5.7%, *P* = 0.004), *Table 1* and *Table S1*. Morbidity and mortality in relation to dose-intensification or second chemotherapeutic drug are reported in *Table S3*.

### Impact of HIPEC regimens and intensification protocols

**Fig. 4:**
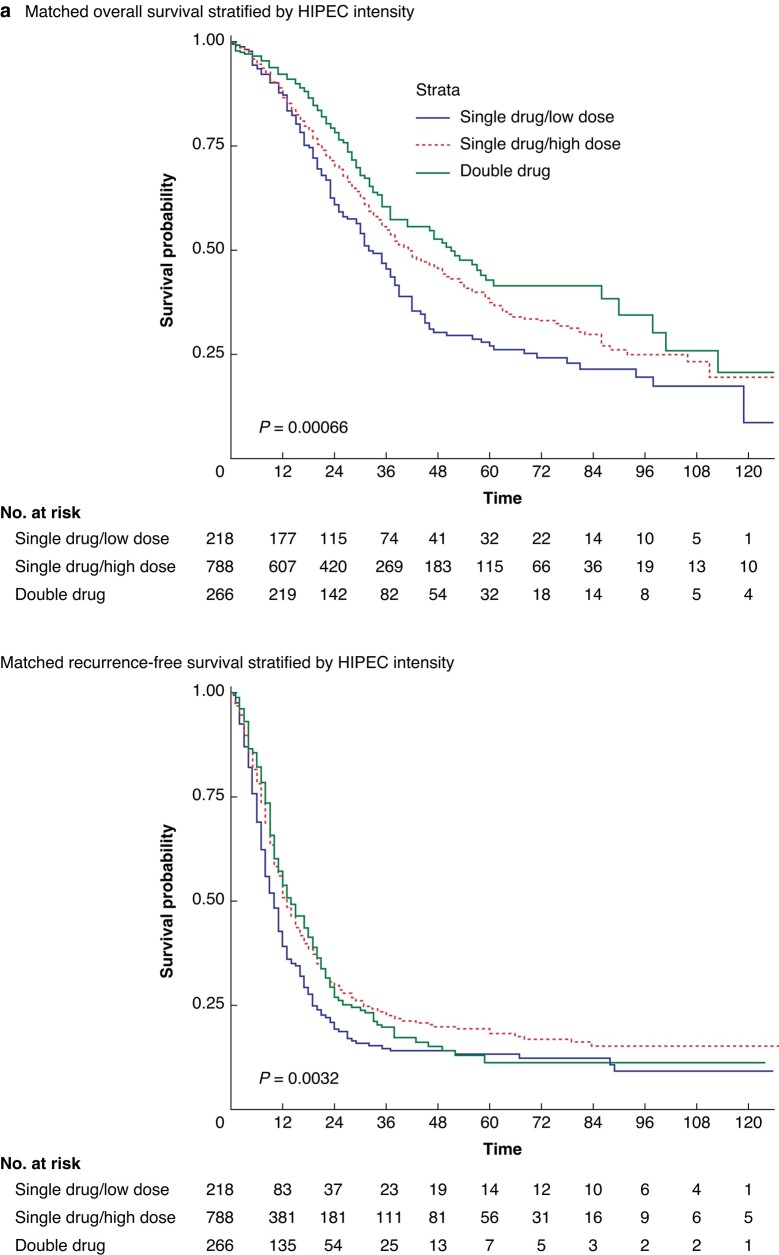

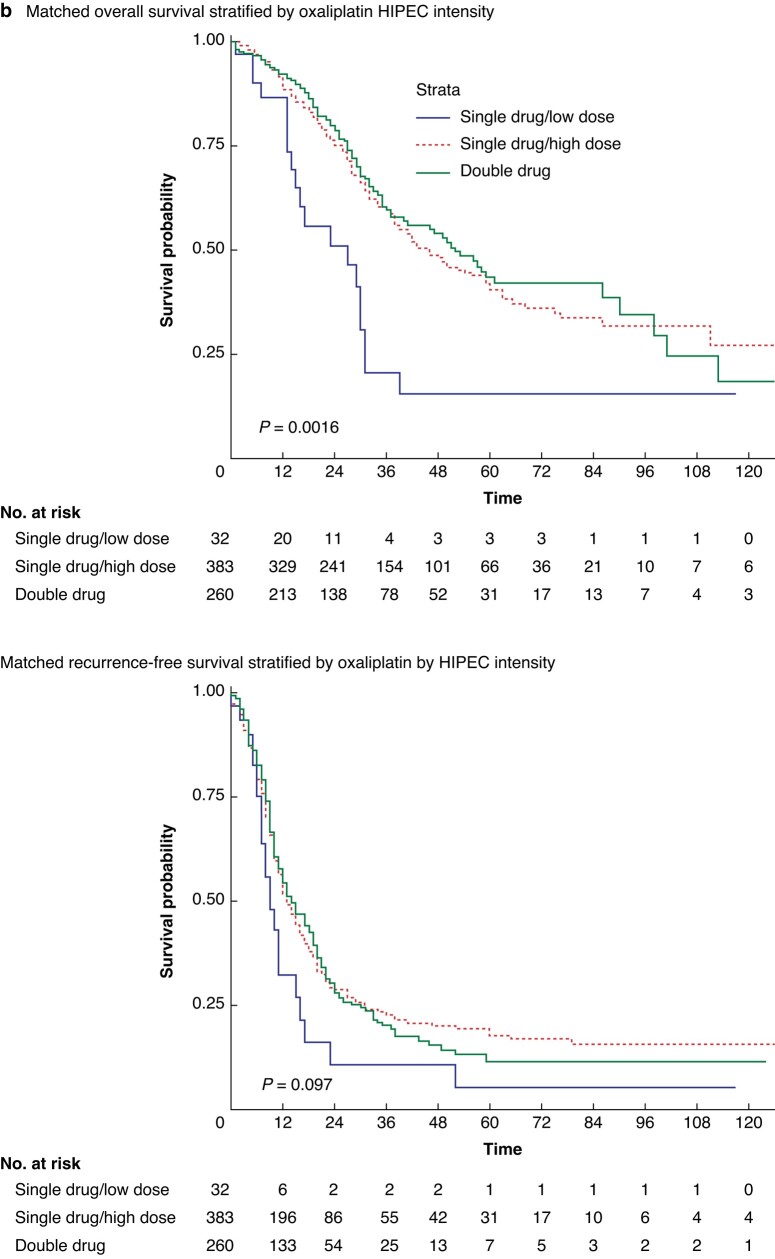

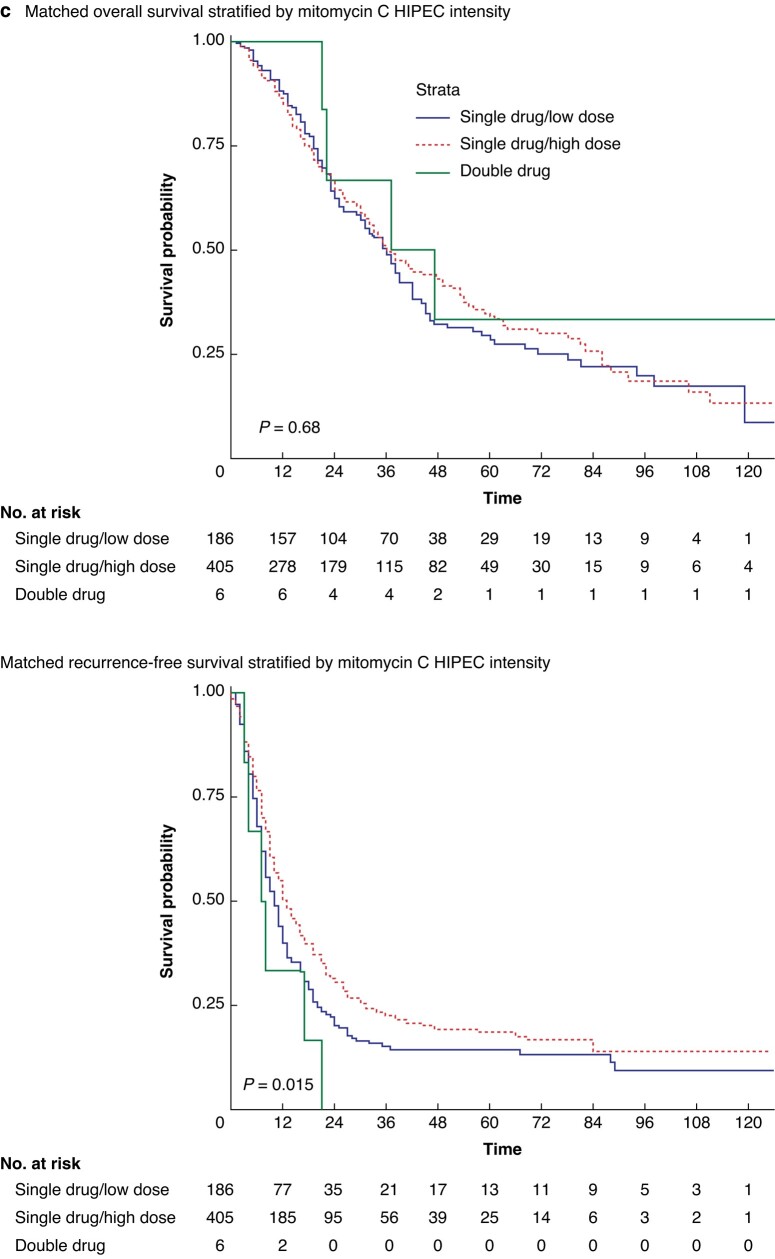
Impact of varying MMC- and Ox-HIPEC protocols on overall survival and recurrence-free survival (continued on next page)

Regarding long-term outcomes, the application of lower doses of HIPEC resulted in significantly worse OS compared to higher doses or double/drug regimens (median 32 months (95% c.i. 29 to 39 months) *versus* 46 months (95% c.i. 42 to 52 months) *versus* 50 months (95% c.i. 41 to 61 months), *P* < 0.001, *Fig. 4*). Similarly, RFS was significantly impacted by HIPEC intensification protocols (median RFS 10 months (95% c.i. 8 to 11 months) *versus* 12 months (95% c.i. 12 to 14 months) *versus* 13 months (95% c.i. 12 to 16 months), *P* = 0.003, *Fig. 4*).

HIPEC, hyperthermic intraperitoneal chemotherapy, MMC, mitomycin C.

Similar results were found for the matched oxaliplatin HIPEC subgroup: median OS 61 months (95% c.i. 51 to 101 months) double-drug HIPEC *versus* 29 months (95% c.i. 17 to 39 months) low-dose single-drug HIPEC *versus* 46 months (95% c.i. 40 to 59 months) high-dose single drug HIPEC, *P* < 0.001, *Fig. 4*. Median RFS for the same groups was as follows: median 15 months (95% c.i. 12 to 19 months) *versus* 10 months (95% c.i. 6 to 17 months) *versus* 13 months (95% c.i. 12 to 15 months), *P* = 0.2, *Fig. 4*.

Equally, single-drug/high-dose MMC protocols showed a borderline significant improved OS compared to either single-drug/low-dose or double-drug MMC-HIPEC protocols (median OS 45 months (95% c.i. 40 to 52 months) *versus* 36 months (95% c.i. 30 to 42 months) *versus* 35 months (95% c.i. 22 to 94 months), *P* = 0.05, respectively, *Fig. 4c*). Similarly, significant differences in RFS were seen depending on MMC-HIPEC protocols (median 12 months (95% c.i. 11 to 14 months) *versus* 10 months (95% c.i. 8 to 12 months) *versus* 8 months (95% c.i. 7 to 14 months), *P* = 0.02, for single-drug/high-dose *versus* single-drug/low-dose *versus* double-drug protocols, respectively, *Fig. 4c*). Upon multivariable analysis, intensification of HIPEC protocols was an independent predictor of OS in unmatched and matched cohorts (*Table 2*), but not for RFS (*Table S2*).

### Impact of treatment time period

A total of 61.8% of the patients in this study were treated in the most recent time period of 2012–2018, with no difference between the groups in terms of proportion treated in this time period—Ox-HIPEC 60.7% *versus* MMC-HIPEC 62.8% (*Table 1*). A trend for lower-dose HIPEC regimens to be used more frequently during the time period of 2001–2009 (23.1%) was documented, but then the proportion of single-drug low-dose regimens went down again in later time periods (2010–2018, 12.4%), whereby the utility of higher-dose single-drug regimens was more frequently used in later decades (52% in 2001–2009, and 62.3% in 2010–2018, *P* < 0.001). Overall survival, however, maintained its difference in this time period (Ox-HIPEC 64 months (95% c.i. 46 months to not reached) *versus* MMC-HIPEC 42 months (95% c.i. 38 to 51 months), *P* = 0.004), *Table S4*. The RFS did not differ here. There was a significant difference in both OS and RFS in the first time period 1991–2001, *Table S4*.

## Discussion

In this global observational study of pmCRC patients who underwent CRS/HIPEC, Ox-HIPEC resulted in improved OS and RFS compared to MMC-HIPEC. Morbidity results were inconclusive, with worse results for MMC-HIPEC, but 90-day mortality was worse for MMC-HIPEC in both analyses. The study found a significant dose–response benefit for both oxaliplatin and MMC-HIPEC, supporting the continued use of HIPEC in colorectal cancer. This stands in contrast to the recent PRODIGE 7 trial^3,9^.

Single-agent low-dose HIPEC was ineffective, but a significant step-wise increase in RFS was observed from low-dose to high-dose to double-agent HIPEC. The observed RFS of 10 months in the low-dose group was similar to the no HIPEC group in the PRODIGE 7 trial (11 months), indicating low doses may not provide any benefit^9^. The RFS increase to 12 and 13 months for the HIPEC intensification groups correlated with the high-dose regimen in PRODIGE 7, but the trial was not powered to show small RFS benefits. The present analysis of over 2000 patients showed a potential RFS benefit with a hazard ratio of 0.84, similar to the benefit seen in PRODIGE 7 (HR 0.90). The focus on RFS instead of OS is because OS is dependent on additional treatments. The congruence between the two studies suggests HIPEC may convey a small but clinically relevant RFS benefit, which likely contributes to the subsequent significant OS benefit seen in the present analysis.

The world primarily uses either oxaliplatin or mitomycin for HIPEC in colorectal cancer^13^. The present study showed that switching from Ox-HIPEC to MMC-HIPEC does not provide any added benefit and may result in added 90-day mortality. The PRODIGE 7 trial used high-dose oxaliplatin HIPEC, but due to the lack of a clear benefit, some institutions switched to MMC-HIPEC. This study suggests the switch is unnecessary. The study found that low-dose MMC-HIPEC (<25 mg/m^2^) may result in worse outcomes than high-dose MMC-HIPEC (>25 mg/m^2^), which achieved similar outcomes to oxaliplatin-based therapies. This is the first study to demonstrate a clear RFS benefit with the high-dose regimen. If a switch has been made, it is advisable to use a high-dose MMC-HIPEC regimen instead of a low-dose regimen, based on a previous preclinical chemosensitivity study^3^.

In a comprehensive review^15^, it was found that 60 HIPEC protocols for pmCRC have been published in 25 years with variations also documented by other systematic reviews^4,16,17^. The present analysis found higher-dose and dual-agent protocols improved OS, particularly if Ox-HIPEC was used. The present study identified oxaliplatin + irinotecan HIPEC as a promising regimen for future trials with a median OS of 61 months and RFS of 15 months—a protocol that has been studied previously with mixed results^15,16^. However, the pronounced survival differences justify a prospective RCT with a reasonable sample size. A randomized trial using this protocol currently underway in Sweden with the aim of starting patient recruitment in 2021^18^.

Two previous cohort studies found no differences in RFS and OS between oxaliplatin- and MMC-HIPEC. One study^19^ reported a RFS of 13.8 *versus* 12.2 months and an OS of 26.5 *versus* 37.1 months in the MMC *versus* oxaliplatin group, respectively. Similarly, another study^20^ found no significant differences in RFS and OS for MMC *versus* oxaliplatin. A large multicentric series from the ASPSM, which included over 500 patients, also showed no differences in OS between oxaliplatin- and MMC-HIPEC^21^. Furthermore, a population-based analysis from the Netherlands found no statistically significant differences in OS, but patients receiving Ox-HIPEC had a 16-month longer OS compared to MMC-HIPEC (46.6 *versus* 30.7 months)^22^. An Australian series showed a significant survival advantage for patients treated with oxaliplatin compared to MMC-HIPEC (56 *versus* 29 months)^23^. However, a systematic review concluded that there was insufficient evidence to support either MMC- or Ox-HIPEC due to differences in treatment protocols^4^ and some authors have found that the outcomes of pmCRC treated with CRS/HIPEC are independent of the intraperitoneal drug^10^. The present analysis found improved overall survival for patients who received Ox-HIPEC compared to those receiving MMC-HIPEC, with a modest and non-significant difference in RFS. This finding is even more relevant when HIPEC is thought of as a locoregional therapy akin to short-course radiotherapy in rectal cancer, and thus the modest and non-significant differences in RFS are considered.

The potential explanation for poor outcomes in patients who received MMC-HIPEC may be due to poor chemosensitivity^3^ or prior poor responses to oxaliplatin-based systemic treatment prior to CRS/HIPEC. This is relevant, as preoperative exposure to systemic oxaliplatin may induce genetic alterations and clonal selections to metastatic deposits^24^. Further, patients with mucinous histopathological subtypes who often metastasize to the peritoneum may not respond well to oxaliplatin-based therapies^17,25,26^. Thus, MMC-HIPEC may be a surrogate for patients with worse tumour biology. Equally, patients who received MMC-HIPEC were less likely to receive adjuvant systemic chemotherapy, which was independently associated with improved OS—albeit this exposure needs to be interpreted with caution due to risk of overadjustment bias and ‘Table 2 fallacy’^14,27^. However, the lack of detailed information on specific regimens of pre- and postoperative systemic chemotherapy is a drawback of the study. Accordingly, it is difficult to conclude a causal relationship between low-dose MMC-HIPEC and worse patient outcomes considering these factors. Double-agent MMC-HIPEC (with cisplatin or irinotecan) was rarely used and no conclusions can be made concerning this subgroup.

There are further limitations to the present study, including selection bias due to its retrospective nature and non-uniform treatment selection criteria across contributing centres, which may lead to a lack of homogeneity in patient populations. Additionally, there are missing data on important prognostic factors (such as the presence of signet ring cells) and it is possible that the differences in patient outcomes are due to uncaptured patient features. Propensity score matching—while a powerful tool for reducing bias in observational studies—can only correct for known variables. Thus, the present study may only provide guidance for reducing HIPEC regimens to high-dose regimens and for evaluating sample sizes for future trials, and it is scientifically unfounded to draw a simple conclusion about the superiority of oxaliplatin-HIPEC. Furthermore, uncertainty remains over how the effect of HIPEC is to be interpreted in the absence of granular data regarding the exact systemic (pre- and postoperative) chemotherapy regimens that were deployed to patients and thus this represents a further shortcoming of the present study.

Despite this, the MitOxHIPEC study has strengths: it is the largest multicentre comparison of HIPEC regimens in pmCRC patients, with over 2000 patients in the unmatched and over 1300 in the matched analyses. To date, the largest series comprises just over 500 patients^21^. Second, collaborating institutions were part of national or international peritoneal surface oncology collaboratives, which maintained prospective databases, reducing missing data. Third, the study used PSM with a 1:1 ratio and nearest-neighbour matching with random order, ensuring an optimal distribution of scores. Finally, the study performed matching on variables known to influence pmCRC patient selection and prognosis and corrected for residual differences in the final modelling to obtain best estimates of the effect of the different HIPEC regimens^28,29^. The methodology potentially offers treatment effect estimation similar to randomized controlled trials^30^.

While the knowledge-base for the utility of HIPEC in peritoneal metastasized colorectal cancer is ever growing, the current body of evidence can be summarized as follows: CRS + MMC-based HIPEC is superior to no CRS/HIPEC^5,31^; neoadjuvant oxaliplatin-based systemic therapy with subsequent CRS + mono-oxaliplatin-based HIPEC is not superior to CRS without HIPEC in patients treated with preoperative oxaliplatin-based systemic chemotherapy (PRODIGE 7^9^); CRS + oxaliplatin may result in better survival than CRS + mitomycin C, particularly if low-dose protocols are used, but if higher-dose MMC regimens are used the outcomes may be equivalent (present study). However, whether CRS + MMC-based HIPEC is better than CRS alone remains unclear and there is no evidence to suggest or refute this treatment regimen, but deploying it to patients remains a leap of faith at present. Thus, with these points above, the present study—while hypothesis generating—helps inform the discussion in this evolving field of medicine and potentially provides a solid basis for future RCTs.

In conclusion, the MitOxHIPEC study suggests that HIPEC has a clinical effect with a dose–response effect, with the oxaliplatin group showing better 90-day mortality rates and high-dose MMC-HIPEC being a worthy alternative. The oxaliplatin + irinotecan regimen showed enhanced efficacy and is promising for future trials. While the data are exploratory, they can inform clinical choices towards standardization in HIPEC use for colorectal cancer.

## Collaborators

Kjersti Flatmark, Wilhelm Graf, Heikki Takala, Andrew M. Lowy, Terence Chua, Joerg Pelz, Dario Baratti, Joel M. Baumgartner, Richard Berri, Pedro Bretcha-Boix, Marcello Deraco, Guillermo Flores-Ayala, Alberto Gomez-Portilla, Santiago González-Moreno, Martin Goodman, Evgenia Halkia, Shigeki Kusamura, Mecker Moller, Guillaume Passot, Marc Pocard, George Salti, Armando Sardi, Maheswari Senthil, John Spilioitis, Juan Torres-Melero, Kiran Turaga, Jean-Marc Bereder, Jean-Louis Bernard, Naoual Bakrin, Sébastien Carrère, Julien Coget, Eddy Cotte, Olivier Facy, Maximiliano Gelli, François-Noël Gilly, Pablo Ortega-Deballon, Guillaume Passot, Patrick Rat, Pascal Rousset, Emilie Thibaudeau, Delphine Vaudoyer.

## Supplementary Material

zrae017_Supplementary_Data

## Data Availability

All de-identified data and analysis code can be made available by contacting the corresponding author, but will require a formal approval process through the research collaborative.
