# Supplementary material for: Hyperthermic intraperitoneal chemotherapy in colorectal cancer
Source: BJS Open. 2024 May 9;8(3):zrae017. doi: 10.1093/bjsopen/zrae017 (PMC11081075; doi:10.1093/bjsopen/zrae017)
Supplement: zrae017_Supplementary_Data [file zrae017_supplementary_data.docx]

**Title**

*Hyperthermic Intraperitoneal Chemotherapy in Colorectal Cancer*

**Authors**

1. Oliver M. Fisher^1, 2, 3^, Chris Brown^4^, Jesus Esquivel^5^, Stein G. Larsen^6^, Winston Liauw^2,7^, Nayef A. Alzahrani^1,8^, David L. Morris^1^, Vahan Kepenekian^9, 10^, Isabelle Sourrouille ^11 ,^ Frédéric Dumont^12,^ Jean-Jacques Tuech^13^, Cécilia Ceribelli^14^, Béranger Doussot^15^, Olivia Sgarbura^16,17^, Mohammed Alhosni^18^, Francois Quenet^17^, Olivier Glehen^9, 10^, Peter H. Cashin^19, 20^

**On behalf of the Peritoneal Surface Oncology Group**

**International (PSOGI), Nordic Peritoneal Oncology Group (NPOG), American Society for Peritoneal Surface Malignancy (ASPSM) and BIG-RENAPE Groups**

^1^ Department of Surgery, St George Hospital, Sydney, Australia

^2^ St George & Sutherland Clinical School, UNSW Australia, Australia

^3^ Notre Dame University School of Medicine, Sydney, Australia

^4^ NHMRC Clinical Trials Centre, Sydney, Australia

^5^ Division of Surgical Oncology, Frederick Memorial Hospital, Frederick, Maryland, United States of America

^6^ Dept. of Surgical Oncology, The Norwegian Radium Hospital, Oslo University Hospital, Sognsvannsveien 20, 0372 Oslo, Norway

^7^ Department of Medical Oncology, St George Hospital, Sydney, Australia

^8^ Department of surgery, National Guard Health Affairs, King Abdulaziz Medical City

Riyadh, Saudi Arabia

^9^ Department of Digestive Surgery, Hôpital Hospitalier Lyon Sud, Hospices Civils de Lyon, Lyon, France

^10^ EA 3738 CICLY, Université Lyon 1, Lyon , France

^11^ Department of Surgery, Institute Gustave Roussy, Villejuif, France

^12^ Department of Oncological Surgery Institut de Cancérologie de l’Ouest René Gauducheau, St Herblain, France

^13^ Department of Digestive Surgery, Centre Hospitalo-Universitaire de Rouen, France

^14^ Department of Surgery Centre Hospitalo-Universitaire l’Archet II, Nice, France

^15^ Department of Digestive Surgery Centre Hospitalo-Universitaire Dijon Bourgogne, Dijon, France

^16^ IRCM, Institut de Recherche en Cancérologie de Montpellier, INSERM U1194, Université de Montpellier, Institut régional du Cancer de Montpellier, Montpellier, F-34298, France.

^17^ Département de Chirurgie Oncologique, Institut régional du Cancer de Montpellier, Montpellier, F-34298, France

^18^ Surgical oncology division, Department of surgery, Sultan Qaboos University Hospital SQUH, Muscat, Oman

^19^ Department of Surgical Sciences, Uppsala University, Uppsala, 75105, Sweden

^20^ Department of Surgery, Akademiska Sjukhuset, 75185 Uppsala, Sweden

**Corresponding author.**

Dr. Oliver M. Fisher, MD, PhD, FMH (Surgery), FRACS, Department of Surgery, Upper Gastrointestinal & Hepatobiliary Surgery, St George Hospital, Sydney, Australia, Email: [ofisher@gmx.ch](mailto:ofisher@gmx.ch)

**Supplementary Materials - Index**

| **Supplementary Methods** |  |
| --- | --- |
| *Details regarding utilised HIPEC protocols* | *pag. 2* |
| *Follow-up* | *pag. 2* |
| *Analysis of Data and Measures to Reduce Potential Confounding* | *pag. 3* |
| *Time period analysis* | *pag. 4* |
| **Supplementary Tables and Figures** |  |
| *Supplementary Table 1 – Unmatched patient demographics* | *pag. 5* |
| *Supplementary Table 2 - Uni & multivariable Cox regression analysis for factors influencing patient recurrence-free survival in unmatched and propensity score matched patient cohorts* | *pag. 6* |
| *Supplementary Table 3 - Clavien-Dindo in-hospital morbidity & 90-day mortality stratified by HIPEC intensification protocols.* | *pag. 7* |
| *Supplementary Table 4 - Overall- and recurrence-free survival outcomes stratified by treatment time-period and utilized HIPEC regimen.* | *pag. 8* |
| *Supplementary Figure S1 – Covariate Balance of Matching Process* | *pag. 9* |
| *Supplementary Figure S2 – Forest plot of effect of HIPEC on OS of different patient subgroups* | *pag. 10* |
| *Supplementary Figure S3 – Forest plot of effect of HIPEC on RFS of different patient subgroups* | *pag.11* |
| **References** | *pag. 12* |

**Supplementary Methods**

*Details regarding utilised HIPEC protocols*

Chemoperfusate was heated to a temperature between 41 and 42.5°C with MMC given at concentrations of a median of 30mg/m^2^ (IQR 12.5–35mg/m^2^), and oxaliplatin given at median doses of 350mg/m^2^ (IQR 300–360mg/m^2^). 5‐fluorouracil 400 mg/m^2^ and leucovorin 50 mg were administered intravenously 1 hour before Ox-HIPEC to potentiate it’s activity. The use of MMC-HIPEC or Ox-HIPEC as a single drug or in combination with another chemotherapeutic agent varied between institutions. In some instances, MMC was combined with either irinotecan or cisplatin, whereas some patients received a combination of oxaliplatin with intraperitoneal irinotecan. To adjust for these variations in HIPEC-protocols, patients were subsequently grouped as follows: Patients receiving single agent MMC at doses <25mg/m^2^ or oxaliplatin <300mg/m2 were labelled as receiving single agent/low-dose HIPEC, and those receiving doses over these thresholds were labelled as receiving single agent/high dose HIPEC. If two agents were used for HIPEC, then these patients were grouped together into a double-drug HIPEC group.

Perfusion duration depended on institutional preference with a median of 90mins (IQR 60–90mins) for MMC and a median of 30mins (IQR 30–30mins) for oxaliplatin. The choice of the main HIPEC drug was largely dependent on institutional preference and in some instances individualized to patient characteristics. For example, in cases where patients had experienced previous platinum-based toxicities, MMC-HIPEC would be used. Preoperative systemic chemotherapy was defined as any pre‐CRS/HIPEC treatment and postoperative adjuvant systemic chemotherapy defined as systemic therapy being administered in the post‐CRS setting.

*Follow-up*

Short-term outcomes of patients were evaluated by grading patient’s postoperative complications according to the Clavien-Dindo classification[1], whereby complications >Grade II were regarded as major complications. All in-hospital deaths were classified as such, but if patients died within 90 days of the date of CRS/HIPEC then these were classified as 90-day mortalities. Follow-up data included the date of last known patient contact and their survival as well as recurrence status at the date of last follow-up. Recurrences were classified as local (peritoneal), local & extraperitoneal or extraperitoneal recurrence according the site of first relapse. Abdominal lymph node and hepatic parenchymal recurrences were classified as extraperitoneal recurrence. Follow‐up included clinical examination, pathology testing, and review of thoraco-abdominopelvic computed tomography scans. All patients were followed up once every 3 months for a minimum of 2 years and once every 6 months thereafter.

*Analysis of Data and Measures to Reduce Potential Confounding*

To correct for potential treatment allocation bias and confounding factors between treatment groups, propensity-score matching was performed to control for patient baseline characteristics.

In a first step, unmatched patient demographics were cross-tabulated and inspected for pre-existing imbalances of preoperative factors that may have impacted the allocation to a particular HIPEC treatment-arm. Subsequently, a propensity score was calculated for each patient as the predicted probability of the allocation to Ox-HIPEC using multivariable logistic regression which included pre- and intraoperative factors identified from the first crude-group comparison supplemented by the addition of factors that may impact patient prognosis including age, gender, American Society for Anaesthesiologists (ASA) category, the present of positive lymph nodes, the presence of liver metastases at the time of CRS/HIPEC, PCI, CC-scores and the use of preoperative systemic chemotherapy. As complete propensity scores cannot be calculated in the presence of missing data for any of the included predicting variables, missing data was computed as such to allow for the lowest attrition rate of study subjects during matching steps. Matching occurred at a 1:1 ratio with no reuse of study subjects using a nearest-neighbor method, and treatment and control units were matched in a random order to ensure optimal distribution of propensity scores. Covariate balance following matching procedures were assessed with Love plots determining mean differences and the difference in cumulative density between groups using the Kolmogorov-Smirnov tests[2].

Overall and RFS times were plotted stratified according the utilised HIPEC drug and associated dose-intensification protocols using the Kaplan-Meier method and resulting survival times compared using the log-rank test. This was first done by analysing crude (unmatched) survival rates and subsequently the matched cohorts.

*Time period analysis*

The impact of the treatment-related time-period was analysed in the matched cohort as the included data spanned a >25-year time-frame. The cohort was divided into four time-periods: 1991-2001, 2002-2006, 2007-2011, and 2012-2018. Exploratory analyses of survival differences were carried out to evaluate whether there was a difference between the groups related to which time period the patient was treated. Median overall and recurrence-free survival was used to compare between Ox-HIPEC and MMC-HIPEC according to each time period. The log-rank test was implemented.

| **Supplemental Table S1. Unmatched patient demographics** | | | | | |
| --- | --- | --- | --- | --- | --- |
| **Variable** |  | **Total (n=2093)** | **Mitomycin C (n=849)** | **Oxaliplatin (n=1244)** | **p-value** |
| **Age** | Median (IQR) | 58.0 (16.0) | 59.0 (15.5) | 56.0 (16.0) | <0.001 |
| **Gender** | Female | 1156 (55.2) | 476 (56.1) | 680 (54.7) | 0.526 |
|  | Male | 937 (44.8) | 373 (43.9) | 564 (45.3) |  |
| **American Society for Anaesthesiologists (ASA) category** | 1 | 214 (10.2) | 54 (6.4) | 160 (12.9) | <0.001 |
|  | 2 | 756 (36.1) | 276 (32.5) | 480 (38.6) |  |
|  | 3 | 137 (6.5) | 63 (7.4) | 74 (5.9) |  |
|  | 4 | 4 (0.2) | 2 (0.2) | 2 (0.2) |  |
|  | Missing | 982 (46.9) | 454 (53.5) | 528 (42.4) |  |
| **Positive lymph nodes** | No | 482 (23.0) | 195 (23.0) | 287 (23.1) | 0.883 |
|  | Yes | 1412 (67.5) | 570 (67.1) | 842 (67.7) |  |
|  | Missing | 199 (9.5) | 84 (9.9) | 115 (9.2) |  |
| **Liver metastasis** | No | 1721 (82.2) | 706 (83.2) | 1015 (81.6) | 0.03 |
|  | Yes | 339 (16.2) | 137 (16.1) | 202 (16.2) |  |
|  | Missing | 33 (1.6) | 6 (0.7) | 27 (2.2) |  |
| **Synchronous vs metachronous disease** | Synchronous | 1050 (50.2) | 407 (47.9) | 643 (51.7) | <0.001 |
|  | Metachronous | 963 (46.0) | 370 (43.6) | 593 (47.7) |  |
|  | Missing | 80 (3.8) | 72 (8.5) | 8 (0.6) |  |
| **Preoperative systemic chemotherapy** | No | 519 (24.8) | 253 (29.8) | 266 (21.4) | <0.001 |
|  | Yes | 1449 (69.2) | 576 (67.8) | 873 (70.2) |  |
|  | Missing | 125 (6.0) | 20 (2.4) | 105 (8.4) |  |
| **Peritoneal cancer index (PCI)** | Median (IQR) | 8.0 (10.0) | 8.0 (9.0) | 8.0 (10.0) | 0.366 |
| **Completeness of cytoreduction (CC) score** | CC-0 | 1896 (90.6) | 763 (89.9) | 1133 (91.1) | 0.254 |
|  | CC-1 | 111 (5.3) | 45 (5.3) | 66 (5.3) |  |
|  | CC-2 | 32 (1.5) | 12 (1.4) | 20 (1.6) |  |
|  | Missing | 54 (2.6) | 29 (3.4) | 25 (2.0) |  |
| **HIPEC intensity** | single drug/low dose | 224 (10.7) | 178 (21.0) | 46 (3.7) | <0.001 |
|  | single drug/high dose | 1077 (51.5) | 451 (53.1) | 626 (50.3) |  |
|  | double drug | 426 (20.4) | 22 (2.6) | 404 (32.5) |  |
|  | Missing | 366 (17.5) | 198 (23.3) | 168 (13.5) |  |
| **Second HIPEC drug** | No second drug | 1426 (68.1) | 722 (85.0) | 704 (56.6) | <0.001 |
|  | Cisplatin | 10 (0.5) | 10 (1.2) | 0 (0.0) |  |
|  | Irinotecan | 416 (19.9) | 12 (1.4) | 404 (32.5) |  |
|  | Missing | 241 (11.5) | 105 (12.4) | 136 (10.9) |  |
| **Perfusion time (minutes)** | Median (IQR) | 30.0 (60.0) | 90.0 (30.0) | 30.0 (0.0) | <0.001 |
| **IV 5-FU during HIPEC** | No | 227 (10.8) | 191 (22.5) | 36 (2.9) | <0.001 |
|  | Yes | 978 (46.7) | 1 (0.1) | 977 (78.5) |  |
|  | Missing | 888 (42.4) | 657 (77.4) | 231 (18.6) |  |
| **Adjuvant systemic chemotherapy** | No | 570 (27.2) | 226 (26.6) | 344 (27.7) | <0.001 |
|  | Yes | 745 (35.6) | 265 (31.2) | 480 (38.6) |  |
|  | Missing | 778 (37.2) | 358 (42.2) | 420 (33.8) |  |
| **Colonic or rectal primary** | Colon | 1659 (79.3) | 677 (79.7) | 982 (78.9) | 0.184 |
|  | Rectum | 174 (8.3) | 60 (7.1) | 114 (9.2) |  |
|  | Missing | 260 (12.4) | 112 (13.2) | 148 (11.9) |  |
| **Signet ring cell pathology** | No | 1035 (49.5) | 413 (48.6) | 622 (50.0) | 0.051 |
|  | Yes | 60 (2.9) | 16 (1.9) | 44 (3.5) |  |
|  | Missing | 998 (47.7) | 420 (49.5) | 578 (46.5) |  |
| **Postoperative complications** | < grade 3-4 | 1116 (53.3) | 420 (49.5) | 696 (55.9) | 0.006 |
|  | >= grade 3-4 | 665 (31.8) | 283 (33.3) | 382 (30.7) |  |
|  | Missing | 312 (14.9) | 146 (17.2) | 166 (13.3) |  |
| **In hospital or 90-day mortality** | No | 1946 (93.0) | 773 (91.0) | 1173 (94.3) | 0.004 |
|  | Yes | 147 (7.0) | 76 (9.0) | 71 (5.7) |  |
| **Operating time (minutes)** | Median (IQR) | 370.0 (180.0) | 373.0 (180.0) | 366.0 (180.0) | 0.409 |
| **Estimated blood loss (mls)** | Median (IQR) | 300.0 (600.0) | 300.0 (615.0) | 300.0 (512.5) | 0.053 |
| **Treatment year** | 1991-2001 | 54 (2.6) | 27 (3.2) | 27 (2.2) | 0.006 |
|  | 2002-2006 | 201 (9.6) | 100 (11.8) | 101 (8.1) |  |
|  | 2007-2011 | 650 (31.1) | 240 (28.3) | 410 (33.0) |  |
|  | 2012-2018 | 1188 (56.8) | 482 (56.8) | 706 (56.8) |  |

| **Supplemental Table S2. Uni & multivariable Cox regression analysis for factors influencing patient recurrence-free survival in unmatched and propensity score matched patient cohorts** | | | | | |
| --- | --- | --- | --- | --- | --- |
|  |  | **Unmatched cohort (n=2093)** | | **Matched cohort (n=1324)** | |
| **Variable** |  | **HR (univariable)** | **HR (multivariable)** | **HR (univariable)** | **HR (multivariable)** |
| **Age** | **Mean (SD)** | 1.00 (0.99-1.00, p=0.399) | 1.00 (0.99-1.00, p=0.239) | 1.00 (0.99-1.00, p=0.394) | 1.00 (0.99-1.00, p=0.478) |
| **Gender** | **Female** | - | - | - | - |
|  | **Male** | 1.11 (0.99-1.24, p=0.080) | 1.07 (0.95-1.20, p=0.245) | 1.07 (0.94-1.22, p=0.291) | 1.05 (0.92-1.20, p=0.480) |
| **Positive lymph nodes** | **No** | - | - | - | - |
|  | **Yes** | 1.45 (1.26-1.66, p<0.001) | 1.46 (1.27-1.68, p<0.001) | 1.45 (1.23-1.70, p<0.001) | 1.44 (1.22-1.70, p<0.001) |
|  | **Missing** | 1.46 (1.17-1.83, p=0.001) | 1.35 (1.08-1.69, p=0.010) | 1.40 (1.09-1.81, p=0.008) | 1.29 (0.99-1.67, p=0.055) |
| **Synchronous vs metachronous disease** | **Synchronous** | - | - | - | - |
|  | **Metachronous** | 1.02 (0.91-1.14, p=0.755) | 1.12 (1.00-1.26, p=0.060) | 1.02 (0.89-1.16, p=0.788) | 1.15 (1.00-1.32, p=0.051) |
|  | **Missing** | 1.02 (0.78-1.34, p=0.858) | 1.28 (0.95-1.72, p=0.103) | 1.19 (0.89-1.59, p=0.250) | 1.38 (1.00-1.91, p=0.053) |
| **Colonic or rectal primary** | **Colon** | - | - | - | - |
|  | **Rectum** | 1.05 (0.87-1.27, p=0.611) | 1.02 (0.84-1.23, p=0.855) | 1.04 (0.84-1.30, p=0.708) | 1.01 (0.80-1.26, p=0.964) |
|  | **Missing** | 0.76 (0.32-1.83, p=0.540) | 0.73 (0.29-1.79, p=0.488) | - | - |
| **Liver metastasis** | **No** | - | - | - | - |
|  | **Yes** | 1.66 (1.43-1.93, p<0.001) | 1.69 (1.44-1.97, p<0.001) | 1.71 (1.43-2.05, p<0.001) | 1.77 (1.47-2.12, p<0.001) |
|  | **Missing** | 2.04 (1.32-3.15, p=0.001) | 2.09 (1.33-3.29, p=0.001) | 2.92 (1.39-6.17, p=0.005) | 2.85 (1.32-6.14, p=0.008) |
| **Preoperative systemic chemotherapy** | **No** | - | - | - | - |
|  | **Yes** | 1.05 (0.92-1.19, p=0.472) | 1.12 (0.98-1.29, p=0.107) | 1.01 (0.88-1.16, p=0.916) | 1.09 (0.94-1.27, p=0.254) |
|  | **Missing** | 0.96 (0.74-1.24, p=0.740) | 1.04 (0.78-1.38, p=0.801) | 0.76 (0.47-1.25, p=0.282) | 0.93 (0.56-1.55, p=0.777) |
| **Peritoneal Cancer Index (PCI)** | **Mean (SD)** | 1.05 (1.04-1.06, p<0.001) | 1.05 (1.04-1.06, p<0.001) | 1.05 (1.04-1.06, p<0.001) | 1.05 (1.04-1.06, p<0.001) |
| **Completeness of cytoreduction (CC) score** | **CC-0** | - | - | - | - |
|  | **CC-1** | 1.62 (1.27-2.08, p<0.001) | 1.23 (0.96-1.59, p=0.107) | 1.67 (1.27-2.20, p<0.001) | 1.26 (0.94-1.67, p=0.116) |
|  | **CC-2** | 4.66 (2.42-8.99, p<0.001) | 6.40 (3.25-12.61, p<0.001) | 4.92 (2.55-9.49, p<0.001) | 6.64 (3.34-13.19, p<0.001) |
|  | **Missing** | 0.77 (0.50-1.20, p=0.248) | 0.95 (0.55-1.66, p=0.862) | 0.71 (0.40-1.25, p=0.235) | 0.87 (0.44-1.72, p=0.688) |
| **HIPEC agent** | **Mitomycin C** | - | - | - | - |
|  | **Oxaliplatin** | 0.92 (0.82-1.03, p=0.144) | 0.89 (0.77-1.02, p=0.092) | 0.86 (0.76-0.98, p=0.020) | 0.84 (0.72-0.98, p=0.025) |
| **HIPEC intensity** | **Single drug/low dose** | - | - | - | - |
|  | **Single drug/high dose** | 0.75 (0.64-0.89, p=0.001) | 0.84 (0.70-1.00, p=0.052) | 0.77 (0.64-0.91, p=0.003) | 0.85 (0.70-1.04, p=0.115) |
|  | **Double drug** | 0.74 (0.62-0.89, p=0.002) | 0.82 (0.65-1.02, p=0.077) | 0.76 (0.61-0.95, p=0.016) | 0.91 (0.70-1.18, p=0.488) |
|  | **Missing** | 0.91 (0.69-1.20, p=0.503) | 0.86 (0.64-1.15, p=0.310) | 0.94 (0.70-1.27, p=0.696) | 0.86 (0.63-1.18, p=0.353) |
| **Postoperative complications** | **< grade 3-4** | - | - | - | - |
|  | **>= grade 3-4** | 1.29 (1.15-1.45, p<0.001) | 1.19 (1.05-1.34, p=0.006) | 1.30 (1.14-1.49, p<0.001) | 1.16 (1.01-1.34, p=0.034) |
|  | **Missing** | 0.73 (0.50-1.07, p=0.106) | 0.81 (0.51-1.29, p=0.378) | 0.72 (0.46-1.11, p=0.133) | 0.81 (0.48-1.37, p=0.434) |
| **Adjuvant Systemic Chemotherapy** | **No** | - | - | - | - |
|  | **Yes** | 0.89 (0.77-1.01, p=0.080) | 0.90 (0.78-1.03, p=0.131) | 0.89 (0.76-1.04, p=0.131) | 0.88 (0.74-1.04, p=0.126) |
|  | **Missing** | 0.93 (0.80-1.07, p=0.289) | 0.87 (0.75-1.02, p=0.096) | 0.97 (0.82-1.14, p=0.695) | 0.86 (0.72-1.03, p=0.099) |

**Supplementary Table S3. Clavien-Dindo in-hospital morbidity & 90-day mortality stratified by HIPEC intensification protocols.**

| **All HIPEC regimens** | | | | | | |
| --- | --- | --- | --- | --- | --- | --- |
|  |  | **Total** | **single drug/low dose** | **single drug/high dose** | **double drug** | **p-value** |
| Postoperative complications | < grade 3-4 | 765 (64.2) | 132 (66.3) | 480 (62.7) | 153 (67.4) | 0.348 |
|  | >= grade 3-4 | 426 (35.8) | 67 (33.7) | 285 (37.3) | 74 (32.6) |  |
| In hospital or 90-day mortality | No | 1126 (94.5) | 194 (97.5) | 717 (93.7) | 215 (94.7) | 0.114 |
|  | Yes | 65 (5.5) | 5 (2.5) | 48 (6.3) | 12 (5.3) |  |
|  |  |  |  |  |  |  |
| **Mitomycin C HIPEC** | | | | | | |
|  |  | **Total** | **single drug/low dose** | **single drug/high dose** | **double drug** | **p-value** |
| Postoperative complications | < grade 3-4 | 351 (62.8) | 110 (65.9) | 234 (63.2) | 7 (31.8) | 0.008 |
|  | >= grade 3-4 | 208 (37.2) | 57 (34.1) | 136 (36.8) | 15 (68.2) |  |
| In hospital or 90-day mortality | No | 517 (92.5) | 163 (97.6) | 333 (90.0) | 21 (95.5) | 0.007 |
|  | Yes | 42 (7.5) | 4 (2.4) | 37 (10.0) | 1 (4.5) |  |
|  |  |  |  |  |  |  |
| **Oxaliplatin HIPEC** | | | | | | |
|  |  | **Total** | **single drug/low dose** | **single drug/high dose** | **double drug** | **p-value** |
| Postoperative complications | < grade 3-4 | 414 (65.5) | 22 (68.8) | 246 (62.3) | 146 (71.2) | 0.085 |
|  | >= grade 3-4 | 218 (34.5) | 10 (31.2) | 149 (37.7) | 59 (28.8) |  |
| In hospital or 90-day mortality | No | 609 (96.4) | 31 (96.9) | 384 (97.2) | 194 (94.6) | 0.274 |
|  | Yes | 23 (3.6) | 1 (3.1) | 11 (2.8) | 11 (5.4) |  |

**Supplementary Table S4. Overall- and recurrence-free survival outcomes stratified by treatment time-period and utilized HIPEC regimen.**

|  | **HIPEC-agent** | | | | **HIPEC-protocol** | | | | | | |
| --- | --- | --- | --- | --- | --- | --- | --- | --- | --- | --- | --- |
| Time period | Ox-HIPEC | MMC-HIPEC | p-value | Ox-HIPEC single drug/low-dose | | Ox-HIPEC single drug/high-dose | Ox-Irinotecan HIPEC (double drug) | MMC-HIPEC single drug/low-dose | MMC-HIPEC single drug/high-dose | MMC-HIPEC double drug | p-value |
| ***1991-2001 (n=41)*** | | | | | | | | | | | |
| Overall survival (median, 95%CI) | 61 (33–NR) | 29 (17–44) | 0.009 | – | | 61 (33–NR) | – | 32 (–) | 26 (17-82) | 29 (12–NR) | 0.006 |
| Recurrence-free survival (median, 95%CI) | 20 (9–NR) | 8 (7–16) | 0.04 | – | | 20 (9–NR) | – | 12 (–) | 10.5 (5–26) | 8 (7–NR) | 0.02 |
| ***2002-2006 (n=130)*** | | | | | | | | | | | |
| Overall survival (median, 95%CI) | 35 (24–90) | 31 (25–37) | 0.1 | 5 (1–NR) | | 27.5 (21–NR) | 54 (32–101) | 32 (18–50) | 31 (26–46) | 15 (4–NR) | <0.001 |
| Recurrence-free survival (median, 95%CI) | 12 (9–24) | 10 (8–13) | 0.2 | 4 (0–NR) | | 10 (3–NR) | 16 (11–14) | 10 (8–24) | 12 (8–15) | 3 (–) | 0.009 |
| ***2007-2011 (n=335)*** | | | | | | | | | | | |
| Overall survival (median, 95%CI) | 40 (35–51) | 45 (39–53) | 0.7 | 30 (23–43) | | 39 (32–56) | 54 (40–NR) | 23 (19–45) | 53 (46–68) | 47 (22–NR) | 0.007 |
| Recurrence-free survival (median, 95%CI) | 14 (12–18) | 13 (11–19) | 0.7 | 10 (6–23) | | 14 (12–17) | 21 (14–28) | 11 (8–20) | 15 (12–23) | 12 (7–NR) | 0.6 |
| ***2012-2018 (n=818)*** | | | | | | | | | | | |
| Overall survival (median, 95%CI) | 65 (46–NR) | 42 (38–51) | 0.004 | 29 (10–NR) | | 60 (42–NR) | MNR (51–NR) | 39 (35–56) | 49 (38–62) | – | 0.04 |
| Recurrence-free survival (median, 95%CI) | 12 (12–15) | 11 (9–12) | 0.1 | 14 (6–NA) | | 12 (11–15) | 13 (11–17) | 9 (7–12) | 12 (10–16) | – | 0.1 |

**Supplementary Figure S1**

**
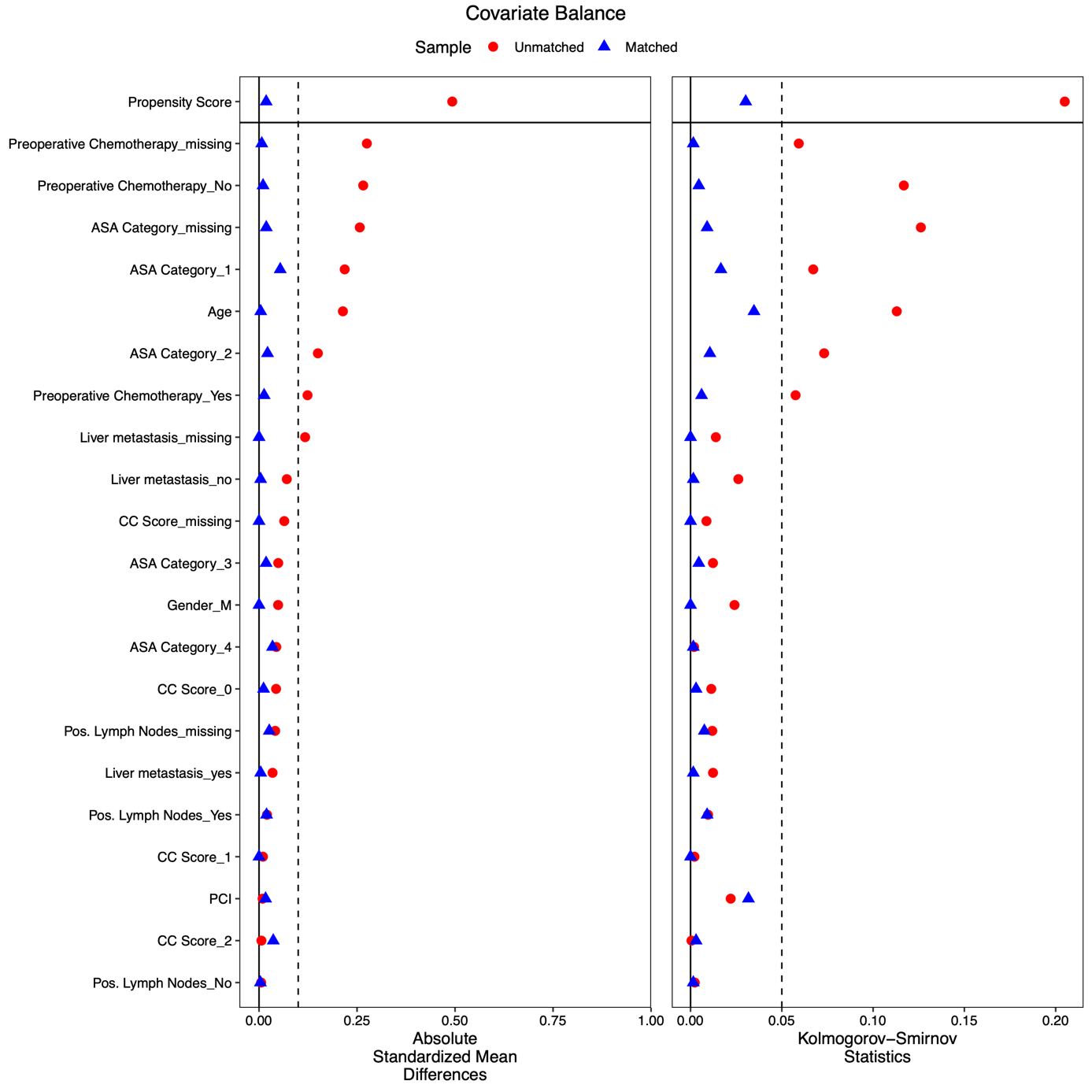
**

**Supplemental Figure S2**


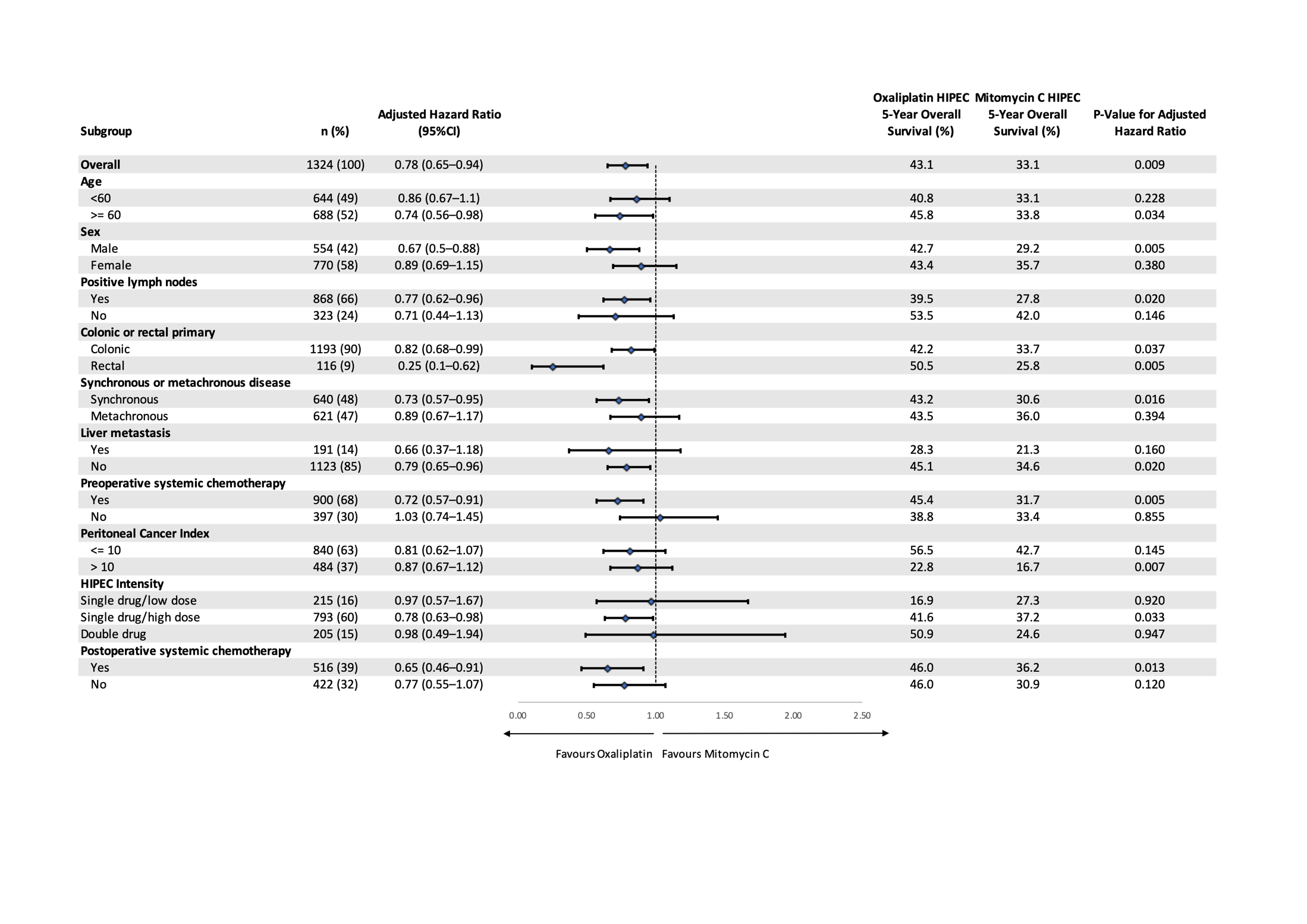


**Supplemental Figure S3**


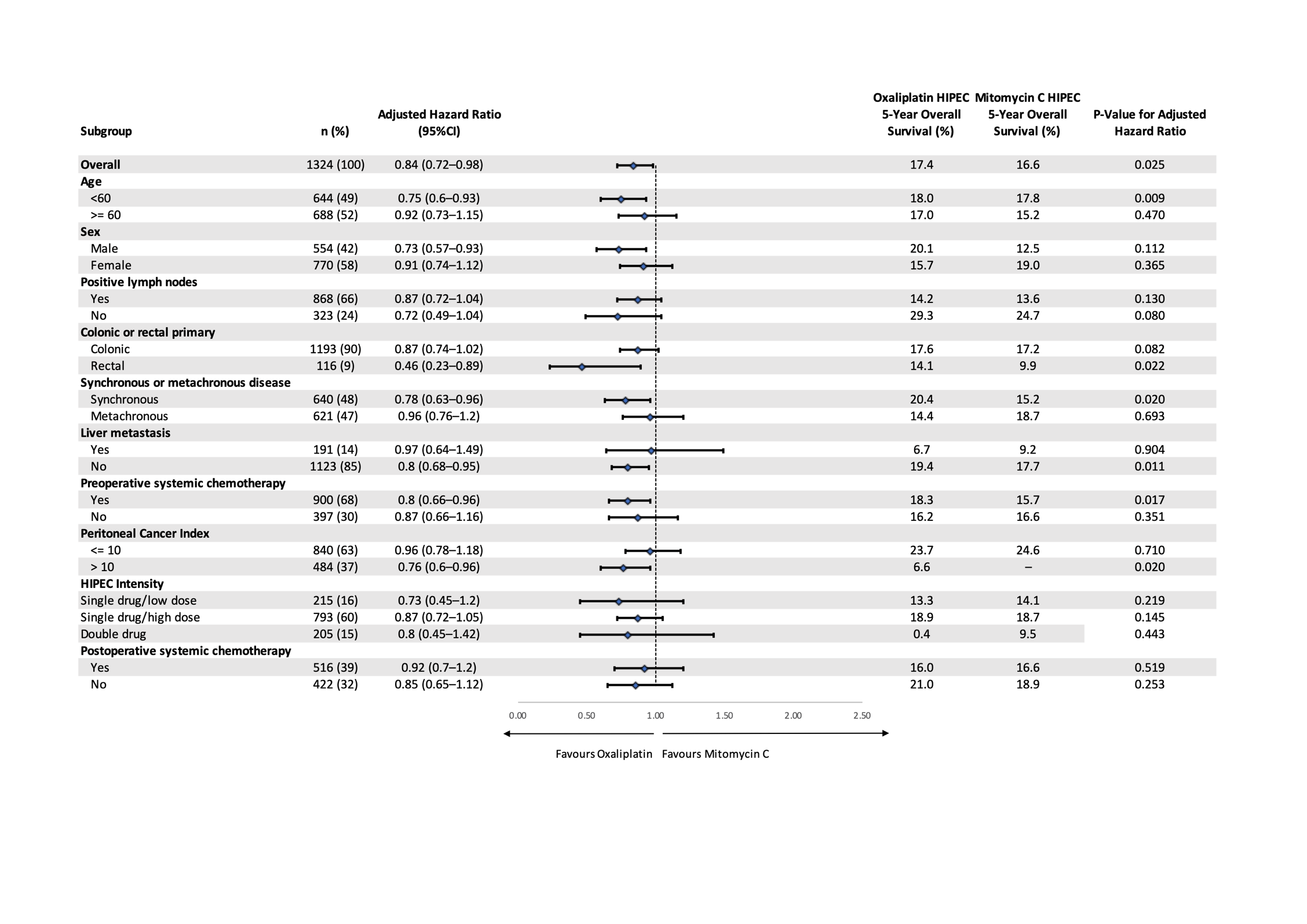


***References***

1. Dindo, D., N. Demartines, and P.A. Clavien, *Classification of surgical complications: a new proposal with evaluation in a cohort of 6336 patients and results of a survey.* Ann Surg, 2004. **240**(2): p. 205-13.

2. Ahmed, A., et al., *Heart failure, chronic diuretic use, and increase in mortality and hospitalization: an observational study using propensity score methods.* Eur Heart J, 2006. **27**(12): p. 1431-9.
